# Devil's Triangle in Kidney Diseases: Oxidative Stress, Mediators, and Inflammation

**DOI:** 10.1155/2012/156286

**Published:** 2012-12-11

**Authors:** Ayşe Balat, Halima Resic, Guido Bellinghieri, Ali Anarat

**Affiliations:** ^1^Division of Pediatric Nephrology, Faculty of Medicine, University of Gaziantep, PK 34, 27310 Gaziantep, Turkey; ^2^Division of Nephrology, Clinical Center University of Sarajevo, Sarajevo, Bosnia and Herzegovina; ^3^Nephrology and Dialysis Unit and Postgraduate School of Nephrology, Messina University, Via Consolare Pompea No. 1867 Ganzirri, 98165 Messina, Italy; ^4^Department of Pediatric Nephrology, Faculty of Medicine, University of Cukurova, 01330 Adana, Turkey

This issue of the International Journal of Nephrology focused on kidney diseases within a devil's triangle, oxidative stress (OS), mediators, inflammation, specifically relating to the clinical significance of identification, and prevention.

Every creature in need of oxygen faces OS. It has a critical role in the molecular mechanisms of renal injury in several kidney diseases, and many complications of these diseases are mediated by OS, mediators, and inflammation. There is a complex relationship between these three; mostly they induce each other. While some of the diseases themselves can contribute to OS, reactive oxygen species (ROS) produced by activated leukocytes and endothelial cells in sites of inflammation cause tissue damage. Although inflammation looks dangerous for the organism, it is a normal reaction of organs and tissues to protect themselves against several invasion(s). It enables the immune system to remove the injurious stimuli and initiate the healing process of tissues. However, the interactions between OS, mediators, and inflammation may result in glomerular damage, proteinuria, electrolyte, and volume instabilities which cause nephron loss, on the long view. Detailed studies on this topic are included in this issue.

The kidney can easily be damaged by ROS, due to the rich structure of long-chain polyunsaturated fatty acids. The article by E. Özbek summarizes the induction of OS within kidney in several conditions, including diabetes, hypertension, hypercholesterolemia, obesity, aging, urinary obstruction, environmental toxins, and molecular mechanisms of these inductions in the light of existing literature data.

Diabetic nephropathy is one of the most common microvascular complications of type 1 and type 2 diabetes mellitus and the leading cause of end-stage renal disease worldwide [1]. 

Rojas-Rivera et al. reviewed the biological bases of oxidative stress and its role especially on diabetic nephropathy, as well as the role of the Keap1-Nrf2 pathway, and recent clinical trials targeting this pathway with bardoxolone methyl, a novel synthetic triterpenoid with antioxidant and anti-inflammatory properties. 

Obesity continues to be a public health problem throughout the world. Epidemiologic studies have shown that 66% of adults and 16% of children and adolescents are overweight or obese [2]. Obesity-related glomerulopathy is an increasing cause of end-stage renal diseases. J. Tang et al. stressed the chronic low-grade systemic inflammation in obesity and discussed the roles of inflammation and oxidative stress in the progression of obesity-related glomerulopathy and possible treatment modalities to prevent kidney injury in obesity, such as the usage of anti-IL-6 receptor antibody, TNF-*α* antagonist, adiponectin, nutritional and surgical interventions to reduce OS.

Hypertension is an another important global health issue both in adults and children. It is one of the major risk factors for the progression of kidney diseases. The relationship between blood pressure and dietary sodium and salt sensitivity has been well known, and renal sodium handling is a key determinant of long-term blood pressure regulation [3]. There is a limited knowledge in the literature regarding the role of ROS-mediated fibrosis and renal proximal tubule sodium reabsorption through the Na/K-ATPase. S. Liu et al. reviewed the possible role of ROS in the regulation of Na/K-ATPase activity. The authors emphasized the importance of further researches whether ROS signaling is a link between the Na/K-ATPase/c-Src cascade and NHE3 regulation and how OS, stimulated by high salt and cardiotonic steroids, regulates Na/K-ATPase/c-Src signaling in renal sodium handling and fibrosis.

Urotensin-II is the most potent mammalian vasoconstrictor identified to date, almost tenfold more potent than endothelin-I [4]. A. Balat and M. Büyükçelik discussed the role of urotensin-II on renal hemodynamics and its possible role on several kidney diseases, such as the minimal change nephrotic syndrome. The article includes a detailed discussion of urotensin-II immunoreactivity in renal biopsy specimens of children with membranoproliferative glomerulonephritis, membranous nephropathy, IgA nephropathy, Henoch-Schonlein nephritis, and focal segmental glomerulosclerosis. Because of its complex relation with OS and other mediators, authors describe it as “more than a mediator” in glomerular diseases. They briefly mention from the effectiveness of U-II antagonism, as a new promising pharmacological treatment target in some kidney diseases.

Given the potential impact of OS, mediators, and inflammation trio, the importance of prevention has come into question. Strong evidence indicates the importance of new molecules that are able to diminish them which in turn may help to decrease the prevalence and/or progression of several kidney diseases. Therefore, further researches are needed to the better understanding of the molecular and clinical mechanisms of this triad. They may help to provide new therapeutical strategies to control several complications in patients with kidney diseases.

*Ayşe Balat* *Halima Resic* *Guido Bellinghieri* *Ali Anarat*
